# Determinants of Postponed Dental Visits Due to Costs: Evidence from the Survey of Health, Ageing, and Retirement in Germany

**DOI:** 10.3390/ijerph16183344

**Published:** 2019-09-10

**Authors:** Ghazal Aarabi, Richelle Valdez, Kristin Spinler, Carolin Walther, Udo Seedorf, Guido Heydecke, Hans-Helmut König, André Hajek

**Affiliations:** 1 Department of Prosthetic Dentistry, Center for Dental and Oral Medicine, University Medical Center Hamburg-Eppendorf, 20246 Hamburg, Germany; 2 Department of Health Economics and Health Services Research, Hamburg Center for Health Economics, University Medical Center Hamburg-Eppendorf, 20246 Hamburg, Germany

**Keywords:** dental visits, dental care, dental health services, health services accessibility

## Abstract

High costs are an important reason patients postpone dental visits, which can lead to serious medical consequences. However, little is known about the determinants of postponing visits due to financial constraints longitudinally. Thus, the purpose of this study was to examine the determinants of postponing dental visits due to costs in older adults in Germany longitudinally. Data from wave 5 and 6 of the Survey of Health, Ageing, and Retirement in Europe was used. The occurrence of postponed dental visits due to costs in the last 12 months served as the outcome measure. Socioeconomic and health-related explanatory variables were included. Conditional fixed effects logistic regression models were used (n = 362). Regressions showed that the likelihood of postponing dental visits due to costs increased with lower age, less chronic disease, and lower income. The outcome measure was neither associated with marital status nor self-rated health. Identifying the factors associated with postponed dental visits due to costs might help to mitigate this challenge. In the long term, this might help to maintain the well-being of older individuals.

## 1. Introduction

Although visiting a dentist for routine check-ups is considered to be an effective means of preventing and treating oral diseases [1], non-attendance occurs frequently [2,3]. Moreover, undetected oral diseases (e.g., untreated caries lesions and periodontitis) continue to be the main reasons for tooth loss [4]. Furthermore, undetected oral diseases can affect a patient’s general well-being [5]. Recent studies have demonstrated that chronic oral inflammations likely affect multiple pathways involved in atherosclerosis [6], atrial fibrillation [7], and chronic kidney disease [8]. Thus, the question of why patients postpone dental visits is relevant.

Research about barriers to dental care has shown that a low level of education [9], male gender [9], low household income (cost for dental treatment) [9,10], and dentate status [11] are reasons for non-attendance at dental appointments [12,13]. However, little is known about the determinants of postponed visits due to financial constraints longitudinally. To date, only two studies have specifically explored the postponement of dental visits due to financial cost.

In addition to increased costs for dental care, income levels and policies linked to specific age groups might be responsible for the inequality in access to dental care [3]. The proportion of people postponing dental visits is greatest among those aged 25–29, slightly elevated among young and middle-aged adults, and low among children and older adults [3]. Interestingly, only 2.2% of European older adults (50+ years) reported not going to the dentist due to costs, and it was demonstrated that cost-related non-attendance was less likely among people who reported having good chewing ability, higher education, and better general health [2]. However, it is unknown whether there are changes over time in individuals, leading them to avoid going to a dentist because of the associated costs. In order to address potential knowledge gaps relevant for health policy, it is necessary to improve our understanding of dental coverage. Furthermore, there is an urgent need to closely evaluate treatment cost-related reasons for dental non-attendance to ensure the affordability, availability, and acceptability of dental care in older adulthood.

Therefore, the present study aimed at investigating the determinants of postponing dental visits due to costs longitudinally in older adults in Germany. Data from waves 5 and 6 of the Survey of Health, Ageing, and Retirement in Europe (SHARE) study were used for explorative data analyses.

## 2. Materials and Methods

### 2.1. Sample

In the current study, longitudinal data were derived from waves 5 (2013) and 6 (2015) of the Survey of Health, Ageing, and Retirement in Europe [14]—a longitudinal, population-based survey. Former waves were excluded from the analysis as the data pertaining to the variables of interest were not available. Our analyses were restricted to Germany. It is worth noting that, using probability samples, community-dwelling individuals of 50 years of age and older and their spouses were interviewed (at home) in European countries and Israel. Individuals were excluded if they were incarcerated, hospitalized, or out of the country during the whole study period, if they were unable to speak the country’s language, or moved to an unknown address. All household members born in 1954 or earlier, were eligible for an interview in wave 1. From wave 2 onwards, for refreshment samples or for new countries entering the survey, there was only one selected individual per household (born in 1956 or earlier in wave 2, 1960 or earlier in wave 4, 1962 or earlier in wave 5, and 1964 or earlier in wave 6). Each wave consisted of a representative sample of non-institutionalized individuals in later life. Drop-off and vignette questionnaires were conducted via paper and pencil, while other data were collected by computer-assisted personal interviews (CAPI). Further details have been provided by Börsch-Supan et al. [14] and Börsch-Supan [15].

During waves 1–4, SHARE was reviewed and approved by the ethics committee of the University of Mannheim several times. Wave 4 of SHARE and the continuation of the project was reviewed and approved by the Ethics Council of the Max Planck Society (most recently in 2018). Oral consent was given prior to the CAPI interview and documented by the interviewers. This consent procedure was approved by the ethics committees. The ethical committee agreed that verbal consent was sufficient and that written consent statements were not necessary to conduct the SHARE interviews. Depending on country-specific legislations, in some of the SHARE countries, the respondents had to sign a written consent statement to be contacted again. Moreover, written consent was necessary for the collection of dried blood spots and for the linking of the SHARE survey data to administrative data.

### 2.2. Outcome Measure: Postponed Visits to the Dentist for Financial Reasons

To quantify postponed visits to the dentist for financial reasons, individuals were asked the following yes-or-no question: “In the last twelve months, to help you keep your living costs down, have you postponed visits to the dentist?”.

### 2.3. Independent Variables

Based on theoretical considerations, independent variables were selected. Age, marital status (married and living together with spouse, registered partnership, married, living separated from spouse, never married, divorced, widowed), and income were used as socioeconomic explanatory variables. With regard to health-related variables, self-rated health was quantified using a single-item measure (1 = excellent, 2 = very good, 3 = good, and 4 = fair, 5 = poor), and morbidity was measured using the number of chronic diseases (heart attack; high blood pressure or hypertension; high blood cholesterol; stroke or cerebral vascular disease; diabetes or high blood sugar; chronic lung disease; arthritis, including osteoarthritis or rheumatism; cancer or malignant tumor; stomach or duodenal ulcer; peptic ulcer; Parkinson’s disease; cataracts; hip fracture; or femoral fracture). Sex and level of education (both constant within individuals over time) were solely used for descriptive purposes. Level of education was quantified using the International Standard Classification of Education [16] (ISCED-97, ranging from 0 (lower level) to 6 (tertiary level)). It is worth emphasizing that time-invariant variables cannot be included as independent variables in fixed effects (FE) regression models (please see the next section).

### 2.4. Statistical Analysis

A main challenge in observational studies is unobserved heterogeneity (e.g., genetic disposition). When these unobserved factors are systematically correlated with the explanatory variables, various estimators (e.g., POLS or random effects regressions) yield inconsistent estimates [17]. FE regressions, in contrast, provide consistent estimates when this correlation is present [17]. FE regressions are not biased by differences in genetic disposition between individuals. The so-called within estimator (FE estimator) relies solely on intraindividual information (changes within units over time). It is worth noting that the conditional FE logistic regressions used in this study only exploit intraindividual information (from waves 5 and 6 of the SHARE study). Consequently, only time-dependent variables can be included in FE regressions. However, time-constant variables can be included as moderating factors (e.g., sex x self-rated health). Cluster-robust standard errors (i.e., standard errors that cluster errors at the individual level) were computed [18]. The P threshold for the determination of statistical significance was 0.05. All statistical analyses were conducted using Stata 15.1 (Stata Corp., College Station, TX, USA). It should be noted that results can solely be generalized to individuals in the population who reported changes in the outcome measure in the period of observation (average treatment effect on the treated). As already argued by Brüderl and Ludwig [19], this reflects the fact that only a small proportion of the population changed their postponement behavior and is therefore not a shortcoming of the FE strategy. The FE strategy has also been used in other studies in the area of epidemiology of health care and community health [20].

## 3. Results

### 3.1. Sample Characteristics

Pooled descriptive characteristics of the observations used in conditional FE logistic regression analysis (n = 362) are described in Table 1. Among these observations, the average age was 63.6 years (SD: ±9.0 years, ranging from 50 to 91 years), 58% were female, and the average self-rated health was 3.6 (±1.0) on a scale ranging from 1 = “excellent” to 5 = “poor”. With regard to education, the majority had an intermediary level of education (61.3%). In total, 57.5% were married and living together with a spouse/registered partnership. The average number of chronic diseases was 1.5 (± 1.4). Further details are displayed in Table 1. In total, 181 individuals (which equals 362 observations) changed their postponement behavior from wave 5 to wave 6 in Germany.

### 3.2. Regression Analysis

The results of conditional FE regression analysis (n = 362) are displayed in Table 2. The pseudo R^2^ value equaled 0.25. Explanatory variables, including age, marital status, income, number of chronic diseases, and self-rated health, showed that the likelihood of postponed dental visits due to financial reasons decreased with increasing age [OR: 0.57, 95% CI: 0.47–0.67], increasing number of chronic diseases [OR: 0.61, 95% CI: 0.43–0.88], and increasing income [OR: 0.99997; 95% CI: 0.99994–0.99999]. The outcome measure was not associated with marital status or self-rated health (Table 2).

## 4. Discussion

### 4.1. Main Findings

The aim of this study was to longitudinally examine determinants of postponed dental visits due to costs in older adults in Germany. Based on SHARE data, the regressions showed that the likelihood of postponed dental visits due to financial reasons decreased with increasing age, increasing number of chronic diseases, and increasing income. The dependent variable was not linked to changes in marital status or changes in self-rated health. In accordance with previous work (e.g., Chen, 2010) translating OR into indices of effect size, the ORs reported in our study are classified as small [21].

### 4.2. Previous Research and Possible Explanations

There is a lack of studies investigating specifically the postponement of dental visits due to cost-related reasons longitudinally [2], and this paper extends our current knowledge on this topic in older adults in Germany.

This study demonstrates that with increasing age, the likelihood of postponing dental visits due to financial reasons decreases. The fifth oral health study recently (2014) demonstrated that individuals aged 65–74 years keep their own teeth longer (on average five teeth more than their counterparts in 1997). Consequently, the increasing number of own teeth allows more individuals in old age the option of a fixed denture [22,23]. Individuals in old age may not postpone their appointment, simply because they depend on the care of dentures and total prosthetics by their dentist. In addition, the demographic change may be leading to a significant increase in the number of old aged individuals [23]. This suggests that our results will become clearer within a few years.

Furthermore, this study observed that postponement of dental visits due to costs was not associated with self-rated general health but instead was associated with having fewer chronic diseases. Interestingly, Listl et al. reported that cost-related non-attendance was associated with worse general health in older adults using the first wave of the SHARE Project [2]. However, it should be noted that this study did not report results on the number of chronic diseases and used aggregated data from 11 European countries in its statistical analyses. The present study observed that lower income is a reason for the postponement of dental care appointments for older adults in Germany. Studies conducted in Canada and Belgium [9,10] found that low income groups, patients without private insurance, and older subjects were more likely to report such barriers. Unfortunately, this problem does not only affect the older generation. A recent study analyzed the Child Public Use File of the 1999 National Survey of America’s Families, including 35,938 children who were younger than 18 years of age [24]. Logistic regression revealed that children from families with low household income did not meet the dental recommendation and had postponed dental care in the last year. Additionally, it is interesting to note that lower income was observed as a barrier to dental care irrespective of whether dental care is supported by compulsory health insurance (i.e., Germany and Belgium) or primarily by privately financed dental care (i.e., Canada and the United States) [25]. The current study demonstrates a social gradient in the postponement of dental visits. Low income represents one out of many factors contributing to socio-economic status (SES). Accumulation of SES factors does not only result in initiation/progression of oral diseases but can also result in postponing dental visits. Therefore, further research should concentrate on the possible determinants of postponement of dental visits due to costs. This may provide information on how to promote responsible dental health utilization behavior at both the individual- and system-level, regardless of the type of dental care system present.

### 4.3. Strengths and Limitations

This study adds first insights into the determinants of postponing dental visits due to financial constraints using a longitudinal approach. The challenge of unobserved heterogeneity was considerably reduced by using FE regressions. Nationally representative data from the well-known SHARE study were used. However, the assessment of postponed dental visits due to financial reasons in the past 12 months was self-reported and thus could be prone to recall bias. However, this recall period is in accordance with previous recommendations [26]. Furthermore, the possibility of reverse causality for the relationship between postponed dental visits and chronic diseases cannot be dismissed. Palgi et al. [27] showed that only a small attrition bias exists in the SHARE data. Although the response rate of SHARE was high compared with other European and recent United States (US) survey studies, the possibility of a small sample selection bias cannot be ruled out.

## 5. Conclusions

Identifying the factors associated with postponing visits to the dentist for financial reasons might help to mitigate this challenge. Visiting the dentist, especially for preventive reasons, is important for not only preventing the development of oral disease but also in order to reduce long-lasting dental costs. On a system level, dental care systems should strive to make costs more transparent and to support prevention campaigns and programs. In the long term, this might help to maintain the well-being of older individuals.

## Figures and Tables

**Table 1:** Characteristics of observations included in conditional fixed effects regression analysis (n = 362; waves 5 and 6; Germany).

	N	%
Sex: Female	210	58.0%
Marital status: married and living together with spouse; registered partnership:	208	57.5%
Education (International Standard Classification of Education; ISCED-97):		
Lower level (0–2)	64	17.7%
Intermediary level (3–4)	222	61.3%
Tertiary level (5–6)	76	21.0%
Self-rated health:		
“Excellent” (1)	13	3.6%
“Very good” (2)	30	8.3%
“Good” (3)	103	28.4%
“Fair” (4)	147	40.6%
“Poor” (5)	69	19.1%
	**Mean**	**SD**
Age in years	63.6	9.0
Household total net income (per year) in Euros	22,376.4	17,706.6
Number of chronic diseases	1.5	1.4
Self-rated health (from 1 = “excellent” to 5 = “poor”)	3.6	1.0

Notes: Please note that time-constant variables (sex and education) were only displayed for descriptive purposes.

**Table 2:** Conditional fixed effects logistic regression models with postponed dental visits for financial reasons as dependent variable (0 = not having postponed dental visits for financial reasons; 1 = having postponed dental visits for financial reasons; waves 5 and 6; Germany).

Independent Variables	Postponed Dental Visits for Financial Reasons
	
Age	0.57 ***
	(0.47–0.67)
Marital status: married and living together with spouse or registered partnership (reference category: other married (living separated from spouse; never married; divorced; widowed))	3.26
	(0.20–52.89)
Income^§^	1.00 *
	(1.00–1.00)
Number of chronic diseases	0.61 **
	(0.43–0.88)
Self-rated health (from 1 = “excellent” to 5 = “poor”)	1.09
	(0.74–1.60)
	
Observations	362
Number of individuals	181
Pseudo R^2^	0.25

Odds ratios and 95% confidence intervals (in parentheses) are given; *** *p* < 0.001, ** *p* < 0.01, * *p* < 0.05; ^§^odds ratio: 0.99997; 95% confidence interval: 0.99994–0.99999.

## References

[1] Afonso-Souza G., Nadanovsky P., Chor D., Faerstein E., Werneck G.L., Lopes C.S. (2007). Association between routine visits for dental checkup and self-perceived oral health in an adult population in Rio de Janeiro: The Pro-Saude Study. Community Dent. Oral Epidemiol..

[2] Listl S. (2016). Cost-related dental non-attendance in older adulthood: Evidence from eleven European countries and Israel. Gerodontology.

[3] Chrisopoulos S., Luzzi L., Brennan D.S. (2013). Trends in dental visiting avoidance due to cost in Australia, 1994 to 2010: An age-period-cohort analysis. BMC Health Serv. Res..

[4] Kassebaum N.J., Bernabe E., Dahiya M., Bhandari B., Murray C.J., Marcenes W. (2014). Global Burden of Severe Tooth Loss: A Systematic Review and Meta-analysis. J. Dent. Res..

[5] Sischo L., Broder H.L. (2011). Oral health-related quality of life: What, why, how, and future implications. J. Dent. Res..

[6] Aarabi G., Heydecke G., Seedorf U. (2018). Roles of Oral Infections in the Pathomechanism of Atherosclerosis. Int. J. Mol. Sci..

[7] Aarabi G., Schnabel R.B., Heydecke G., Seedorf U. (2018). Potential Impact of Oral Inflammations on Cardiac Functions and Atrial Fibrillation. Biomolecules.

[8] Akar H., Akar G.C., Carrero J.J., Stenvinkel P., Lindholm B. (2011). Systemic consequences of poor oral health in chronic kidney disease patients. Clin. J. Am. Soc. Nephrol..

[9] Kengne Talla P., Gagnon M.P., Dramaix M., Leveque A. (2013). Barriers to dental visits in Belgium: A secondary analysis of the 2004 National Health Interview Survey. J. Public Health Dent..

[10] Locker D., Maggirias J., Quinonez C. (2011). Income, dental insurance coverage, and financial barriers to dental care among Canadian adults. J. Public Health Dent..

[11] Macek M.D., Cohen L.A., Reid B.C., Manski R.J. (2004). Dental visits among older U.S. adults, 1999: The roles of dentition status and cost. J. Am. Dent. Assoc. (1939).

[12] Hakeberg M., Wide Boman U. (2017). Dental care attendance and refrainment from dental care among adults. Acta Odontol. Scand..

[13] Molete M.P., Yengopal V., Moorman J. (2014). Oral health needs and barriers to accessing care among the elderly in Johannesburg. SADJ J. South Afr. Dent. Assoc./Tydskrif van die Suid-Afrikaanse Tandheelkundige Vereniging.

[14] Börsch-Supan A., Brugiavini A., Jürges H., Kapteyn A., Mackenbach J., Siegrist J., Weber G. (2008). First results from the Survey of Health, Ageing and Retirement in Europe (2004–2007). Starting the Longitudinal Dimension.

[15] Börsch-Supan A., Jürges H., Börsch-Supan A., Jürges H. (2005). The Survey of Health, Ageing and Retirement in Europe—Methodology.

[16] UNESCO (1997). International Standard Classification of Education; ISCED.

[17] Cameron A.C., Trivedi P.K. (2005). Microeconometrics: Methods and Applications.

[18] Stock J.H., Watson M.W. (2008). Heteroskedasticity-Robust Standard Errors for Fixed Effects Panel Data Regression. Econometrica.

[19] Brüderl J., Ludwig V., Wolf C. (2015). Fixed-effects panel regression. The Sage Handbook of Regression Analysis and Causal Inference.

[20] Hajek A., König H.H. (2018). Which factors lead to frequent attendance in the outpatient sector among individuals in the second half of life? Evidence from a population-based longitudinal study in Germany. BMC Health Serv. Res..

[21] Chen H., Cohen P., Chen S. (2010). How Big is a Big Odds Ratio? Interpreting the Magnitudes of Odds Ratios in Epidemiological Studies. Commun. Stat. Simul. Comput..

[22] Jordan R.A., Bodechtel C., Hertrampf K., Hoffmann T., Kocher T., Nitschke I., Schiffner U., Stark H., Zimmer S., Micheelis W. (2014). The Fifth German Oral Health Study (Funfte Deutsche Mundgesundheitsstudie, DMS V)—Rationale, design, and methods. BMC Oral Health.

[23] Jordan R. (2016). Fünfte Deutsche Mundgesundheitsstudie (DMS V).

[24] Yu S.M., Bellamy H.A., Kogan M.D., Dunbar J.L., Schwalberg R.H., Schuster M.A. (2002). Factors that influence receipt of recommended preventive pediatric health and dental care. Pediatrics.

[25] The Health Systems and Policy Monitor. https://www.hspm.org/mainpage.aspx.

[26] Bhandari A., Wagner T. (2006). Self-reported utilization of health care services: Improving measurement and accuracy. Med. Care Res. Rev..

[27] Palgi Y., Shrira A., Zaslavsky O. (2015). Quality of life attenuates age-related decline in functional status of older adults. Qual. Life Res. Int. J. Qual. Life Asp. Treat. Care Rehabil..

